# Gliridae and Eomyidae (Rodentia) of the Miocene Tagay fauna (Olkhon Island, Lake Baikal, Eastern Siberia)

**DOI:** 10.1007/s12549-022-00551-1

**Published:** 2022-11-18

**Authors:** Gudrun Daxner-Höck, Thomas Mörs, Ivan A. Filinov, Alexander A. Shchetnikov, Baatarjav Bayarmaa, Oljuna Namzalova, Margarita A. Erbajeva

**Affiliations:** 1 Rupertusstr. 16, 5201 Seekirchen, Austria; 2 Natural History Museum Vienna, Burgring 7, 1010 Vienna, Austria; 3 Department of Palaeobiology, Swedish Museum of Natural History, P.O. Box 50007, 10405 Stockholm, SE Sweden; 4 Bolin Centre for Climate Research, Stockholm University, Stockholm, Sweden; 5 Institute of the Earth’s Crust, Siberian Branch, Russian Academy of Sciences, Lermontova str. 128, Irkutsk, 664033 Russia; 6 Geological Institute, Russian Academy of Sciences, Pyzhevsky lane 7, Moscow, 119017 Russia; 7 Laboratory of Geoarchaeology of Baikal Siberia, Irkutsk State University, 5 Armii str. 52, Irkutsk, 664025 Russia; 8 A.P. Vinogradov Institute of Geochemistry, Siberian Branch, Russian Academy of Sciences, Favorskogo str. 1a, Irkutsk, 664033 Russia; 9 Institute of Paleontology and Geology, Mongolian Academy of Sciences, S. Danzan Street –3/1, P.O.B.46/650, Ulaanbaatar, 15160 Mongolia; 10 Dobretsov Geological Institute, Siberian Branch, Russian Academy of Sciences, Sahianova Str. 6a, Ulan-Ude, 670047 Russia

**Keywords:** Siberia, Lake Baikal, Miocene, Rodentia, Systematics, Methods

## Abstract

The **s**mall mammals Myomiminae indet. (Gliridae), *Leptodontomys* cf*. gansus* Zheng and Li, 1982 (Eomyidae) and the new species *Keramidomys sibiricus* nov. spec*.* (Eomyidae) are described. They were collected from six layers of the middle to upper part of the Tagay-1 section on Olkhon Island. The glirid Myomiminae indet. is represented by only a few isolated teeth, the small eomyid *Leptodontomys* cf*. gansus* by a mandible with two teeth, and the second small eomyid *Keramidomys sibiricus* nov. spec*.* by several isolated teeth and a mandible. The ancestral tooth characteristics of *Keramidomys sibiricus* nov. spec*.* indicate an early evolutionary stage of *Keramidomys* in Asia. The suggested age of the assemblage is Early/Middle Miocene transition.

## Introduction

The field campaign 2014 at the Tagay locality (Olkhon Island, Lake Baikal) aimed at investigating the sediment sequence of the Tagay Formation (Mats et al. 2011) along section Tagay-1 by means of geology, sedimentology, lithology, palaeontology, magnetostratigraphy and geochemistry. The fossils studied here were collected during two field campaigns at the Tagay locality. One part was collected by A. Kossler in the 1990s and comprises gastropods, fish-teeth and remains of frogs, turtles, lizards and mammals (Kossler 2003; Daxner-Höck et al. 2013). The second part was collected by Erbajeva and her team in 2014 and it also contains remains of gastropods, ectothermic vertebrates and mammals, mainly isolated teeth of rodents, lagomorphs and insectivores. The palaeontological focus of the field campaign 2014 was on the small mammal record and possible evidences of mammal evolution in the course of deposition of the Tagay sediments.

Though almost all sediment layers of the Tagay-1 section were investigated from the bottom to the top, only the layers 11, 10, 9, 7, 6, 5 and 3 of the upper part of the section (at meters 6–2 down from the top level) yielded fossils. In this paper we focus on the rodent families Gliridae and Eomyidae. They were recovered from four individual layers of the section, layers 9, 7, 6 and 5 (Daxner-Höck et al. 2022, this issue: fig. 3).

## Material and methods

**Fig. 1:**
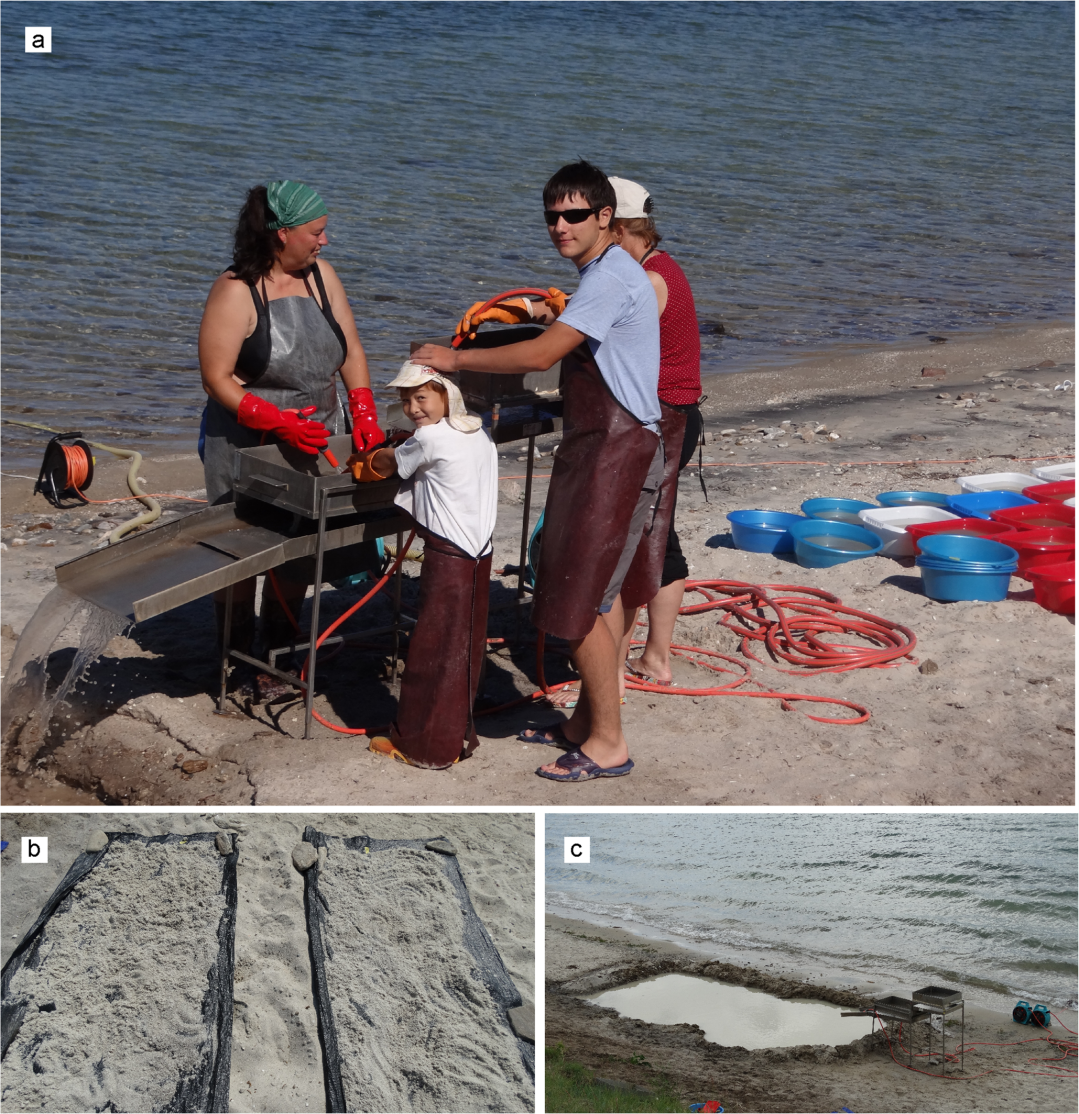
The wet-screening equipment: washing table, sieve sets and hoses (**a**), black textile tarpaulins for drying samples (**b**), settling basin to clarify the muddy water after wet-screening of samples (**c**). Photos by the authors.

**Fig. 2:**
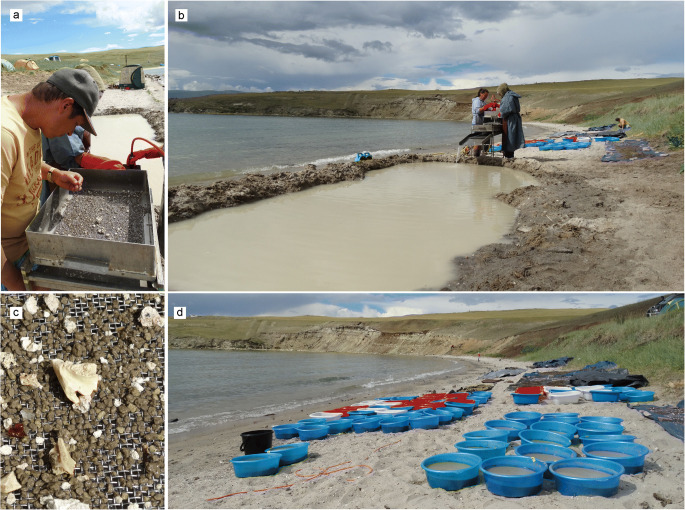
Wet screening process at Lake Baikal (**b**). In the uppermost coarse sieve larger bone fragments were found (**a, c**). Before wet-screening the dried sediment will be soaked in water (**d**). Photos by the authors.

**Fig. 3:**
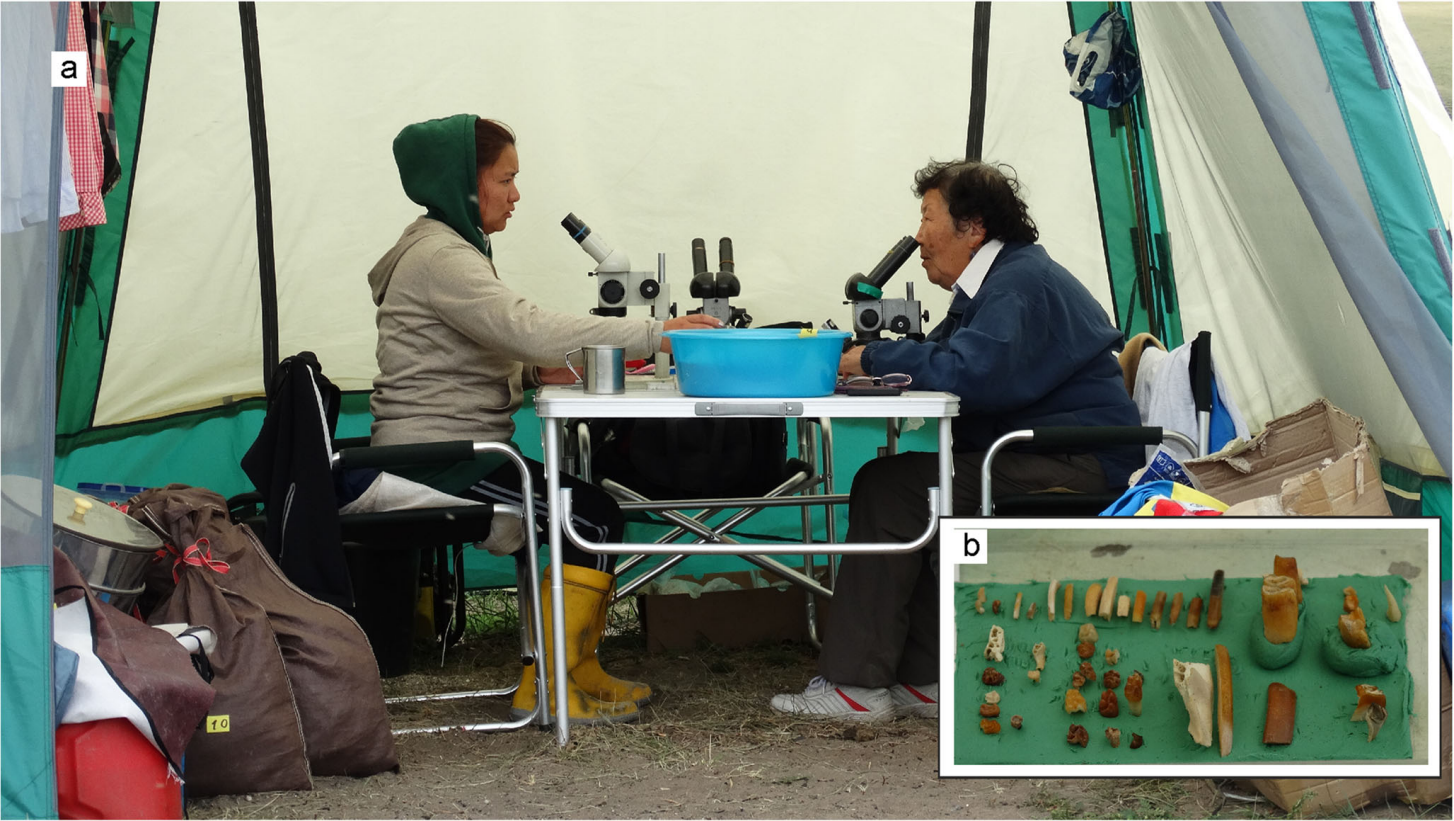
Selecting fossils from the residuals of wet-screened sediment by using stereo-microscopes (**a**). Partial fossil output from layer 9 (**b**). Photos by the authors.

The method of wet-screening of sediment samples in place was used at the Tagay locality in order to optimise collecting of small mammal fossils during the field campaign 2014 (Figs 1-3). The method was established in Europe by Dutch colleagues in the 1960ies and since then used all over Europe, Turkey, Pakistan, China, and Mongolia, mainly in places far away from any laboratory facilities. The wet- screening equipment consists of a divisible washing table with sieve sets of 0.5, 2.5 and 5.0 mm mesh sizes, a water pump and hoses (Fig. 1). The palaeontological work started with layer-by-layer collecting of test samples along the Tagay-1 section, each sample of approximately 20 kg. From beds with positive fossil record we collected additional samples. The total amount of investigated sediment was ~ 2000 kg. In order to optimise the wet-screening process, the collected sediment was dried on black textile tarpaulins, then soaked in bowls with water for 10–30 minutes (Fig. 2). The relation of sediment and water was 1:4. After soaking, the sediment-mush was washed through the sieve-set under flowing water, then the muddy water was clarified in the settling basin before flowing back to the lake. The residuals of washed samples were dried again, and the fossils picked out by using head-lenses and stereo-microscopes (Fig. 3).

The fossils collected by Kossler in the 1990s are housed in three collections: The small mammal remains are integrated in the collection of the Natural History Museum, Geological-Paleontological Department, Vienna (NHMW), the ectothermic vertebrates are deposited in the Bavarian State Collection for Paleontology and Geologie in Munich (BSPG), and the gastropods in the collection of the Freie Universität Berlin, Section of Paleontology. After publication all fossils collected during the field-campaign in 2014 by M. Erbajeva and her team will be stored in the collection of the Zoological Institute of Russian Academy of Sciences (ZIN), St. Petersburg.

SEM images were made by Daniela Gruber [Core Facility Cell Imaging and Ultrastructure Research, University of Vienna - member of the Vienna Life-Science Instruments (VLSI)] by using the Scanning Electron Microscope (SEM) JEOL IT 300 LV LaB6 and Sputter coater JEOL JFC-2300HR.

**Fig. 4:**
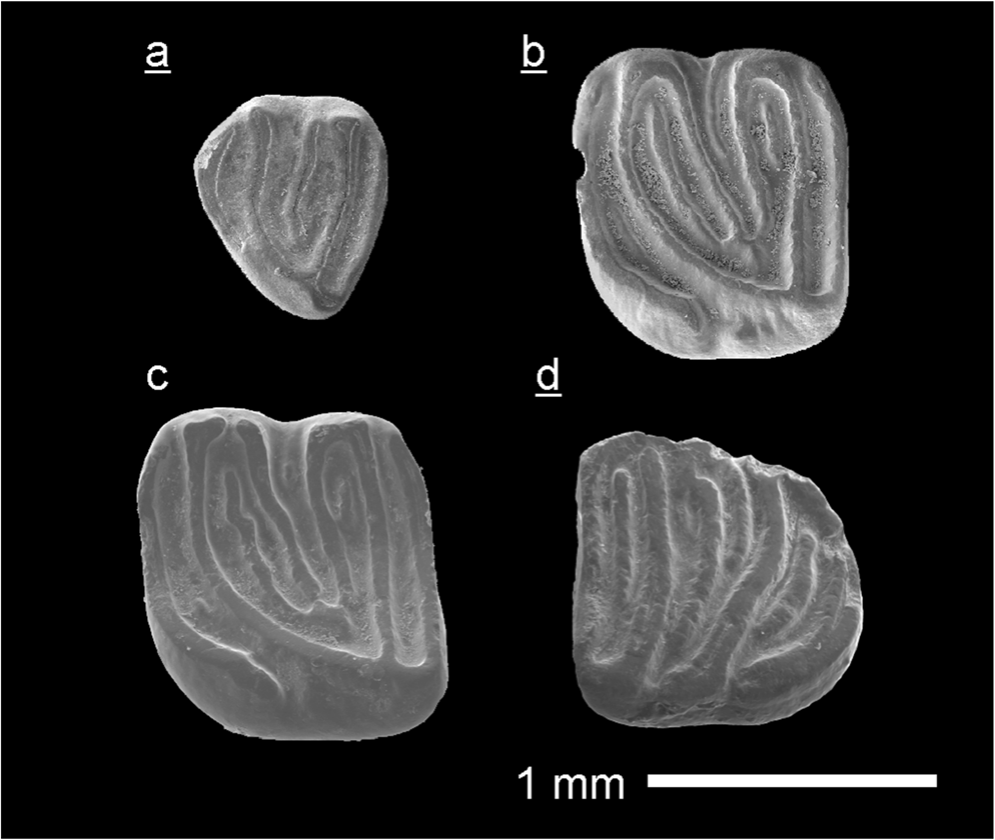
Myomiminae indet. from the Tagay-1 section (layers 9, 7 and 5) of the locality Tagay (Olkhon Island, Baikal region, Siberia) and from test sample Kossler (Ko) of the same locality. All teeth are figured in occlusal view. **a** Right D4 (NHMW2009/0070/0001), Ko. **b** Right M1/2 (NHMW2009/0070/0002), Ko. **c** Left M1/2 (ZIN 106452), layer 9. **d** Right m3 (ZIN 106453), layer 5. (underlined = right, not underlined = left).

To facilitate comparisons, all right-side teeth are figured as mirror images (as if they were left ones), and their figure letters are underlined (e.g. Fig. 4b shows a right M1/2). All measurements are given in mm.

For classification above genus level we follow McKenna and Bell (1998). Description of teeth follows the terminology of Gliridae (Daams 1981) and Eomyidae (Engesser 1990).

## Systematic Palaeontology

Class Mammalia Linnaeus, 1758

Order Rodentia Bowdich, 1821

Family Gliridae Muirhead, 1819

Subfamily Myomiminae Daams, 1981

Myomiminae indet.

(Fig. 4, Tab. 1)

**Table 1:** Measurements of *Myomiminae indet*. teeth from the Tagay-1 section (layers 9, 7 and 5) of the locality Tagay (Olkhon Island, Baikal region, Siberia) and from test sample Kossler (Ko) of the same locality.

Myomiminae indet.					
object	coll. number	length	width	Tagay-1	Fig.
M1 left	ZIN 106452	1.02	1.18	layer 9	4c
m3 right	ZIN 106453	1.00	1.02	layer 5	4d
p4 right fragm.	ZIN 106454	--	--	layer 7	
D4 right	NHMW2009/0070/0001	0,71	0.81	Ko	4a
M1 right	NHMW2009/0070/0002	1.07	1.19	Ko	4b

2013: *Miodyromys* sp. – Daxner-Höck, Böhme and Kossler: 511, Plate 22.1, figs. 6–7.

**Locality, Stratigraphy:** Tagay-1 section (layers 9, 7, 5; samples 2014) and test sample Kossler (Ko) from Olkhon Island, Baikal Region, Siberia; Tagay Formation (Mats et al. 2011); Lower/Middle Miocene transition.

**Material** (Tab. 1): Three teeth (ZIN 106452–106454) of layers 9, 7 and 5 of the Tagay-1 section collected by Erbajeva and colleagues 2014, and two teeth (NHMW 2009/0070/0001–0002) collected by Kossler in the 1990s from the identical Tagay locality (Kossler 2003; Daxner-Höck, Böhme and Kossler 2013: 511, Plate 22.1, figs. 6–7).

### Description

The Gliridae teeth belong to a very small myomimine species. The main characteristics are as follows. Small size, low tooth crowns and complex dental pattern. Upper molars miss the entoloph and have concave occlusal surface. The lingual wall of upper molars is ornamented. All cheek teeth have numerous loph(id)s of almost equal elevation and thickness. The loph(id)s are separated by rather narrow valleys.

**D4** (Fig. 4a) is of triangular outline, and very low crowned. It has five transverse lophs of equal thickness. The anteroloph is of medium length and isolated from paracone and protoloph. The protoloph is long, it connects with the long metaloph. The long posteroloph has a lingual connection with the protocone and a weak labial connection with the metaloph. The posterior centroloph is of medium length, it has free labial and lingual ends. No roots are preserved.

**M1/2** (Fig. 4b, 4c): There are two specimens of similar size and shape, a right M1/2 (Fig. 4b) and a left M1/2 (Fig. 4c). The occlusal outline is almost square (slightly wider than long). The teeth have six main transverse ridges (the anteroloph, protoloph, anterior and posterior centrolophs, metaloph and posteroloph) and three extra ridges (the anterior and posterior extra ridges and a third one between the centrolophs). The lophs and extra ridges are almost equal in elevation and thickness. The long isolated anteroloph extends along the anterior margin from labial to lingual. At the lingual margin it turns towards posterior, however, it does not connect with the protoloph.

The oblique protoloph extends toward its lingual connection with the metaloph. The metaloph and the posteroloph have lingual connections with the protocone. The posteroloph has no labial connection with the metaloph. The anterior centroloph is longer than the posterior centroloph but does not contact the entoloph. The posterior centroloph is labially fused with the metacone and the metaloph. The two available M1/2 slightly differ by: the presence (right M1/2) or absence (left M1/2) of the labial fusion of protoloph, paracone and anterior centroloph and by the length of extra ridges. The anterior extra ridge is longer than the posterior extra ridge. The lingual wall of M1/2 is ornamented. The M1/2 have three roots (one lingual, two labial).

**p4:** The available p4 fragment shows a long anterior root and the anterior part of the crown, i.e. the trigonid and part of the talonid. The trigonid shows the anterolophid fused with the centrolophid forming a circle. The preserved part of the talonid is composed of the posterolophid lingually fused with the entoconid and mesolophid. Labially the mesolophid turns forward and fuses with the anterolophid-centrolphid circle.

**m3** (Fig. 4d): The occlusal outline is triangular with rounded corners, with a maximal width in its anterior part and narrowing towards the posterior. The m3 has five main lophids (anterolophid, metalophid, centrolophid, mesolophid and posterolophid) and three extra ridges (the anterior and posterior extra ridges, and a third extra ridge between centrolophid and mesolophid). The lophids and extra ridges are almost equal in elevation and thickness, but differ in length. The labial ends of metalophid, mesolophid and posterolophid turn forward. There are loose contacts of anterolophid and metalophid, and mesolophid and posterolophid. There is no lingual entolophid nor any distinct lingual lophid-connections, however, the anterolophid, metalophid and centrolophid have loose lingual contacts, also the mesolophid and posterolophid. The anterior extra ridge is the longest among the extra ridges, the posterior extra ridge is of medium length, and the extra ridge between centrolophid and mesolophid is shortest. No roots are preserved.

### Discussion

The described teeth from Tagay are in best agreement with the dental features of the subfamily Myomiminae. We follow the classification of Daams and De Bruijn (1995) and their opinion about origin and diversification of the family Gliridae. The fossil record suggests an European, early Eocene origin. Dormice underwent their first diversification during the Eocene and Oligocene, a diversity peak in the Early Miocene, and a decline during the Middle and Late Miocene. The main centre of diversification and distribution was Europe (Daams and De Bruijn 1995). From there Gliridae dispersed to Asia and Africa.

The Miocene record of Gliridae in Central and East Asia is very poor in comparison with Europe and Turkey. It is restricted to a few genera, e.g. *Miodyromys* Kretzoi, 1943, *Myomimus* Ognev, 1924, *Orientiglis* Qiu and Li, 2016 and *Microdyromys* De Bruijn, 1966 known from China (Wu 1985; Wu 1986; Qiu and Li 2016), and *Miodyromys* Kretzoi, 1943, *Microdyromys* De Bruijn, 1966 and *Prodryomys* Mayr, 1979 from Kazakhstan (Kowalski and Shevyreva 1997). Except for *Orientiglis* (only known from China) the above listed Gliridae genera originated in Europe.

The few glirid teeth from Tagay show some morphological similiarities with species of the genera *Miodyromys* Kretzoi, 1943, *Vasseuromys* Baudelot and Bonis de, 1966 and *Orientiglis.* The Tagay specimens are of similar size with *Vasseuromys elegans* Wu, 1993 (age: Early Miocene, MN3–4; Germany; Wu 1993) and *Orientiglis wuae* (Qiu,^1996^) (ranging in China from the Early to the Late Miocene; Qiu and Li 2016), and are in the lower size range of *Miodyromys asiamediae* Maridet et al. 2011 (in China ranging from the Early to Late Miocene; Qiu and Li 2016). However, reliable genus and species determination of the Tagay glirid is not possible because of the unknown variation of dental morphology and size. Consequently, we describe the Siberian dormouse as Myomiminae indet.

Family Eomyidae Winge, 1887

Genus *Leptodontomys* Shotwell, 1956

*Leptodontomys* cf. *gansus* Zheng and Li, 1982

(Fig. 5)

2013: *Eomyops oppligeri* – Daxner-Höck, Böhme and Kossler: 511–512, Plate 22.1, fig. 16.
2020: *Eomyops oppligeri* Engesser, 1990 – Kimura, Casanovas-Vilar, Maridet, Kalthoff, Mörs and Tomida: 187.

**Locality, Stratigraphy**: Tagay-1 section (layer 7; sample 2014) and test sample Kossler (Ko) from Olkhon Island, Baikal Region, Siberia; Tagay Formation (Mats et al. 2011); Lower/Middle Miocene transition.

**Material and measurements:** A fragmentary right mandible with i and m1 (ZIN 106446) from layer 7 of the Tagay-1 section; alveolar length of p4–m3 = 3.34 mm; m1 length = 0.82 mm, m1 width = 0.80 mm.

Left m1/2 (NHMW2009/0072/0001) from test sample Kossler (Daxner-Höck et al. 2013:511-512, Plate 22.1, fig. 16); m1/2 length = 0.85 mm, m1/2 width = 0.80 mm.

### Description

**Fig. 5:**
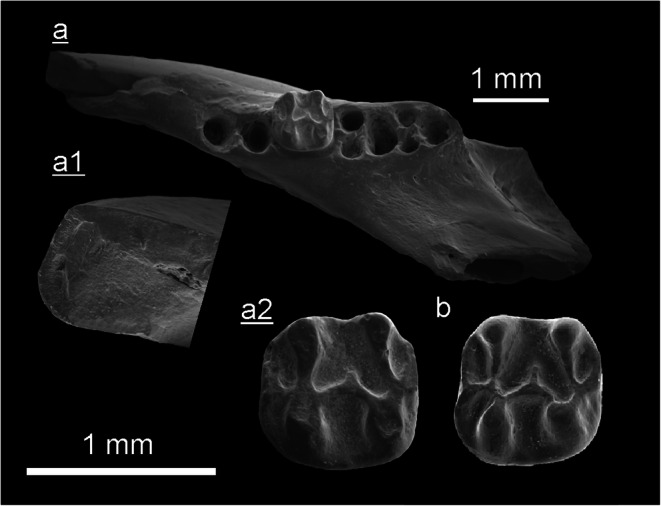
*Leptodontomys* cf. *gansus* Zheng and Li, 1982 from the Tagay-1 section (layer 7) of the locality Tagay (Olkhon Island, Baikal region, Siberia) and from test sample Kossler (Ko) of the same locality. All teeth are figured in occlusal view. **a** Fragmentary right mandible with m1 and i (ZIN 106446), layer 7. **a1** Right incisor of (ZIN 106446). **a2** Right m1 of (ZIN 106446). **b** Left m1/2 (coll. NHMW 2009/0072/0001), Ko. (underlined = right, not underlined = left).

**Mandible:** The fragmentary right mandible (ZIN 106446; Fig. 5a) displays m1 and a fragmentary lower incisor. The alveolar view shows that p4 has two roots (one anterior and one posterior) and m1-3 have three roots (two anterior and one posterior). The masseteric fossa extends to below p4, and the mental foramen is placed ahead of the mandibular toothrow. The mandible is rather slender.

#### Teeth

The described teeth are brachyodont, bunodont, and range among the smallest Eomyidae teeth known from Europe and Asia.

**i** (ZIN 106446; Fig. 5a1) has a smooth anterior surface without enamel ridges.

**m1** (ZIN 106446; Fig. 5a2) and m1/2 (NHMW2009/0072/0001; Fig. 5 b) are rectangular in occlusal outline, the corners are rounded. The four main conids are in opposite positions.

The anterolophid displays a lingual and labial branch and connects the metalophid and the anterior arm of the protoconid, respectively. The transverse metalophid attaches to the metaconid and to the protoconid (Fig. 5a2), or to the anterior arm of protoconid (Fig. 5 b), respectively. The slightly backwards directed hypolophid attaches to the posterior arm of the hypoconid and to the posterolophid, respectively. A very short mesolophid arises from the slightly convex longitudinal crest. There are four lingual synclinids: the narrow 1^st^ synclinid between the lingual anterolophid and the metalophid; the wide 2^nd^ and 3^rd^ synclinids are continuous; and the narrow 4^th^ synclinid is enclosed by the posterolophid, hypolophid and entoconid.

The lower molars have three labial sinusids: the deep anterior sinusid (enclosed by the labial anterolophid and the protoconid), the wide symmetrical sinusid, and a shallow posterior sinusid (located posterior to the right-angled connection of posterolophid and posterior hypoconid arm).

### Discussion

The bunodont Eomyidae teeth from the Tagay-1 section (Siberia) are very small. The tooth morphology resembles two Eomyidae genera, *Leptodontomys* Shotwell, 1956 and *Eomyops* Engesser, 1979, likewise. *Leptodontomys* was originally described from the Late Miocene of North America (Shotwell 1956), later the name was also used for European occurrences (Hartenberger 1967; Hugueney and Mein 1968; Fahlbusch 1970, 1973, 1979). To separate the European *Leptodontomys* species from their North American counterparts, Engesser (1979) erected the genus *Eomyops*. He described a “crenulated lower incisor” as “the most important difference between *Leptodontomys* from North America and Europe” and figured the labial side of a fragmented lower incisor with two parallel enamel ridges from the French locality La Grive, labelled as *Eomyops* aff. *catalaunicus* (Engesser 1979:27 and fig. 8b). However, Kalthoff et al. (2022) state that there is no indication that the figured lower incisor is from a toothed mandible, and consider it as an isolated tooth specimen and as such not safely identified. They assume that Engesser (1979) misinterpreted the description of the lower incisor of *Leptodontomys catalaunicus* in Hugueney and Mein (1968:196) having two ridges although the authors described only one longitudinal edge (“…une bosse bien marquée du côté externe de la branche…”). Judging from the faunal list from de Bruijn et al. (1992) and descriptions of lower incisor ornamentation in Kalthoff (2006), Engesser’s two-ridged lower incisor from La Grive originated from either *Cricetodon, Hispanomys, Eumyarion*, or *Anomalomys*. Later, based on the figured two-ridged incisor fragment in Engesser (1979), Ruiz-Sanchez et al. (2009) attributed an isolated, two-ridged incisor from Morteral-20A to their new taxon *Eomyops noeliae*. This incisor almost certainly represents *Eumyarion* as judged from the faunal list of Morteral-20A given by Ruiz-Sanchez et al. (2009). According to Kalthoff et al. (2022), no eomyid taxon features a two-ridged lower incisor ornamentation. Based on their study, consisting of incisor material from toothed mandibles, Kalthoff et al. (2022) state with certainty that both North American *Leptodontomys* and Eurasian *Leptodontomys*/*Eomyops* have one longitudinal edge, although it is rather shallow in the former and well developed in the latter. In consequence, outer enamel ornamentation with two enamel ridges as a distinctive character between the North American *Leptodontomys* and the Eurasian *Leptodontomys/Eomyops* no longer holds. As the cheek teeth of both genera are remarkably similar in morphology, the validity of *Eomyops* has been questioned by several authors and the genera may be synonymous (Qiu 1994; de Bruijn et al. 2012; Kimura et al. 2020).

The five European „*Eomyops“* species are: “*E.*” *noeliae* Ruiz-Sanchez, Calatayud and Freudenthal, 2009 (Early Miocene, lower Aragonian), the large sized “*E*.” *hebeiseni* Kälin, 1997 (early Middle Miocene, MN5), the smal “*E.*” *oppligeri* Engesser, 1990 (Middle Miocene, MN7/8), and the medium sized “*E.*” *catalaunicus* (Hartenberger, 1967) (Late Miocene to Pliocene, MN9–MN14) and “*E.*” *bodvanus* (Janossy, 1972) (Pliocene, MN14).

Aside of the North American *Leptodontomys* and European (“*Eomyops*”) occurrences, very small bunodont Eomyidae teeth with *Leptodontomys*/“*Eomyops”* pattern were found in several localities in China, predominantly from Nei Mongol (Inner Mongolia). These fossils were attributed to the genus *Leptodontomys* (Zheng and Li 1982; Qiu and Li 2016). The Chinese species are: the smaller *L. gansus* Zheng and Li, 1982 and the larger *L. lii* Qiu and Li, 2016 both ranging from Early to Late Miocene (Chinese Mammal Ages/Stages Shanwangian to Baodean; Kimura et al. 2020). The Early and Middle Miocene fossil record of *L. gansus* is very poor, only two teeth are described from Gashunyinadege (Early Miocene, Shanwangian) and two teeth from loc. 346 (Middle Miocene, Tunggurian), however, rather rich material is known from Balunhalagen, Ertemte 2 and other Chinese localities (Late Miocene, Bahean to Baodean) of Nei Mongol in Northern China (Qiu and Li 2016).

The first find from Siberia (Tagay locality), a single, very small bunodont Eomyidae tooth (m1/2), was described as *E. oppligeri* (Daxner-Höck et al. 2013). Its molar size and pattern is similar with “*E.*” *oppligeri* and *L. gansus*, likewise. The new mandible with incisor and m1, excavated from the same locality in the course of the field campaign 2014 supports *Leptodontomys* rather than “*Eomyops*” because of the slender mandibular ramus and the smooth anterior surface of the incisor without enamel ridges. Hence, the Siberian species is described as *L.* cf. *gansus*, and the former description as “*Eomyops oppligeri*” is synonymous, however, the fossil material is not adequate for reliable species identification.

Genus *Keramidomys* Hartenberger, 1967

*Keramidomys sibiricus* nov. spec*.*

(Fig. 6, Tab. 2)

**Fig. 6:**
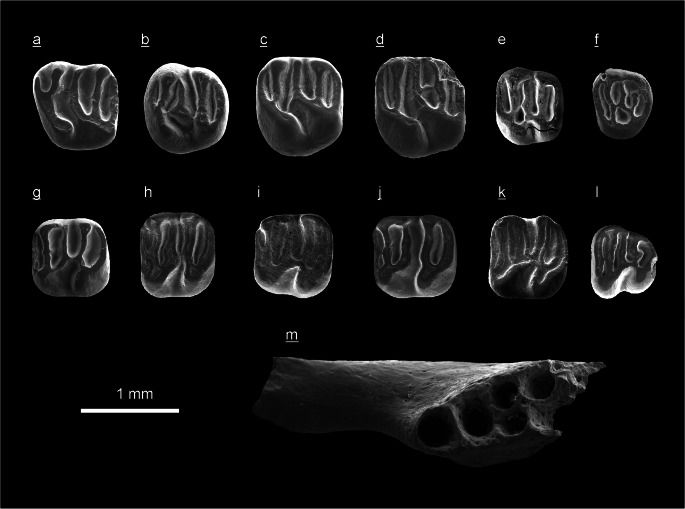
*Keramidomys sibiricus* nov. spec. of the locality Tagay (Olkhon Island, Baikal region, Siberia), from Tagay-1 section (layers 9, 7 and 6) and from test sample Kossler (Ko) of the same locality. All teeth are figured in occlusal view. **a** Right D4, paratype (ZIN 106448), layer 7. **b** Right P4, paratype (ZIN 106450), layer 6. **c** Right M1, holotype, (ZIN 106447), layer 7. **d** Right M1, paratype (ZIN 106449), layer 6. **e** Left M2 (NHMW2009/0071/0001), Ko. **f** Right M3 (NHMW2009/0071/0002), Ko. **g** Right m1/2. **h** Left m1/2 (NHMW2009/0071/0003), Ko. **i** Left m1/2 (NHMW2009/0071/0004), Ko. **j** Right m1/2 (NHMW2009/0071/0007), Ko. **k** Right m1/2 (NHMW2009/0071/0008), Ko. **l** Left m3 (NHMW2009/0071/0005), Ko. **m** Right mandible frag. (ZIN 106447), layer 9. (underlined = right, not underlined = left).

**Table 2:** Measurements of *Keramidomys sibiricus* nov. spec. teeth of the locality Tagay (Olkhon Island, Baikal region, Siberia), from Tagay-1 section (layers 7 and 6) and from test sample Kossler (Ko) of the same locality.

*Keramidomys sibiricus* nov. spec*.*					
object	coll. number	length	width	Tagay-1	Fig.
D4 right (P)	ZIN 106448	0.86	0.88	layer 7	6a
M1 right (H)	ZIN 106447	0.90	1.00	layer 7	6c
M1 right (P)	ZIN 106449	0.90	1.02	layer 6	6d
P4 right (P)	ZIN 106450	0.93	0.93	layer 6	6b
M2 left	NHMW2009/0071/0001	0.76	0.90	Ko	6e
M3 right	NHMW2009/0071/0002	0.69	0.83	Ko	6f
m1/2 left	NHMW2009/0071/0003	0.83	0.93	Ko	6h
m1/2 left	NHMW2009/0071/0004	0.83	0.90	Ko	6i
m1/2 right	NHMW2009/0071/0006	0.83	0.86	Ko	6g
m1/2 right	NHMW2009/0071/0007	0.88	0.93	Ko	6j
m1/2 right	NHMW2009/0071/0008	0.83	0.90	Ko	6k
m3 left	NHMW2009/0071/0005	0.76	0.86	Ko	6l
m3 right	NHMW2009/0071/0009	0.74	0.79	Ko	
1 fragm. m1/2	NHMW2009/0071/0010	–	–	Ko

**Fig. 7:**
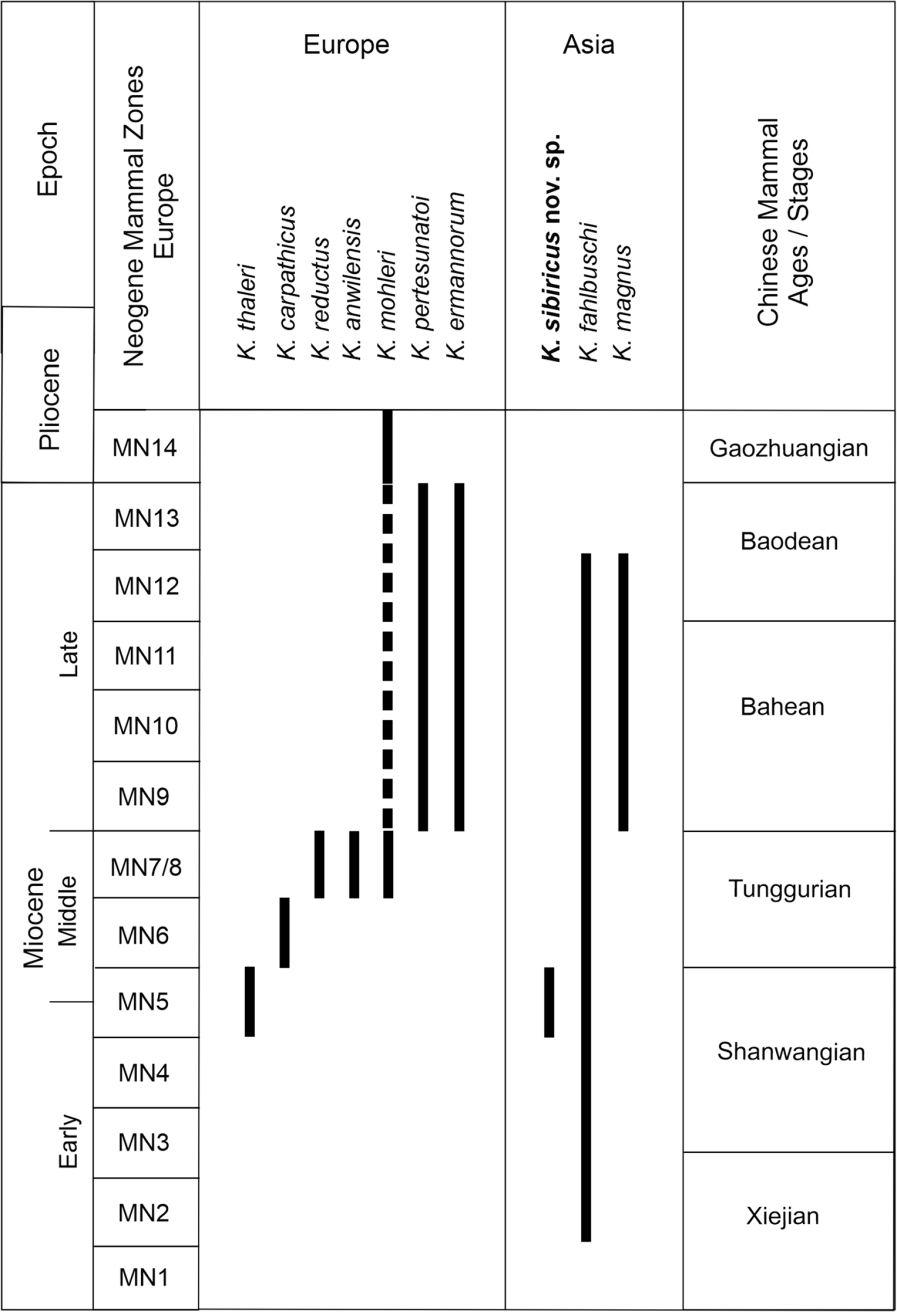
Stratigraphic ranges of *Keramidomys* species in Europe (Engesser 1990; Daxner-Höck 1998; Daxner-Höck and Höck 2009; Mein 2009) and China (Qiu and Li 2016; Kimura et al. 2020).

2013: *Keramidomys* aff. *mohleri* Engesser, 1972 vel *Keramidomys* aff. *fahlbuschi* Qiu, 1996 – Daxner-Höck, Böhme and Kossler: 511–512, Plate 22.1, figs. 8, 9, 10, 11, 12, 13, 14, 15.

**Etymology:** According to the first discoveries of *Keramidomys* in Siberia.

**Holotype** (H): Right M1 (ZIN 106447; Fig. 6c) from layer 7 of the Tagay-1 section.

**Paratypes** (P): A fragmentary right mandible without teeth (ZIN 106451; Fig. 6m) from layer 9, and three cheek teeth (ZIN 106448–106450) from layers 7 and 6 (Tab. 2). All type specimens were collected at the Tagay-1 section by Erbajeva and colleagues in 2014.

**Additional material:** Ten cheek teeth (NHMW2009/0071/0001–NHMW2009/0071/0010; Tab. 2). These specimens were collected by Kossler (Ko) in the 1990s (Kossler 2003; Daxner-Höck et al. 2013: 511–512, Plate 22.1, figs. 8 - 15) at the Tagay locality.

**Type locality and stratigraphy:** Locality Tagay, Tagay-1 section (layers 9, 7, 6), from Olkhon Island, Baikal Region, Siberia; Tagay Formation (Mats et al. 2011); Lower/Middle Miocene transition.

**Diagnosis:** The teeth of *K. sibiricus* nov. spec. show a strong trend toward lophodonty, however, the main cusp(id)s are not completely incorporated into the loph(id)s. The teeth are low crowned, with a slightly concave occlusal surface, by upper teeth having higher labial cusps, lower teeth higher lingual cuspids than loph(id)s, respectively. Five long transverse loph(id)s, a sinus(id) and four synclines/synclinids are characteristic of upper and lower teeth, respectively. Presence of anteroloph and 1^st^ syncline of P4. Hence, the anteroloph and 1^st^ syncline are present in all upper teeth. The anterolophid of lower molars is weak, mostly consists of a labial and a lingual branch. The molars are almost square in occlusal outline, but slightly wider than long, and have rounded corners. The upper molars are larger than the lower ones. D4 and P4 are almost as large as M1. M2 and M3 are smaller. All upper teeth have three roots, p4 has two roots, m1-2 have four roots and m3 has three roots. The Chinese species *K. fahlbuschi* Qiu, 1996 shares with *K. sibiricus* nov. spec. the rather shallow sinus, the main cones/conids incompletely merging into the lophs, and the medium sizes of M2–3 and m1–3.

**Differential diagnosis**: The dental pattern is largely in agreement with the genus *Keramidomys*, however, *K. sibiricus* nov. spec. shows some characteristics reminiscent of the ancestral genus *Asianeomys* Wu et al., 2006. These primordial characteristics are: drop shaped cone(id)s incompletely merged into loph(id)s, and the presence of anteroloph and 1^st^ syncline of P4. The specific P4 features of *K. sibiricus* nov. spec. sporadically can be observed in *K. fahlbuschi* Qiu, 1996 (China) and *K. thaleri* Hugueney and Mein, 1968 (Europe), but are absent in all other *Keramidomys* species.

Differing from *K. sibiricus* nov. spec. the European species *K. thaleri* Hugueney and Mein, 1968, *K. carpathicus* (Schaub and Zapfe, 1953), *K. reductus* Bolliger, 1992, *K. pertesunatoi* (Hartenberger, 1967), *K. anwilensis* Engesser, 1972 and *K. ermannorum* Daxner-Höck and Höck, 2009 are smaller and show variable modifications of dental structures, i.e. reduction of number and length of loph(id)s, and changes of their direction. Of these species *K. thaleri* and *K. carpathicus* are smallest, i.e. significantly smaller than *K. sibiricus* nov. spec.

*K. mohleri* Engesser, 1972 is of comparable size with *K. sibiricus* nov. spec., however, it differs by strong lophodonty, the deep sinus(id), the incomplete longitudinal crest of upper and lower molars, respectively, and by the much smaller P4 without anteroloph and 1^st^ syncline.

*K. fahlbuschi* differs from *K. sibiricus* nov. spec. by: the smaller P4 and M1, the weak or absent anteroloph and 1^st^ syncline of P4.

*K. magnus* Qiu and Li, 2016 (also from China) differs from *K. sibiricus* nov. spec. by the strong lophodonty, i.e. cusp(id)s completely merged into the loph(id)s, it also differs by narrow valleys, the P4 without anteroloph and 1^st^ syncline, the deep sinus(id).

### Description of the holotype (Fig. 6c)

The occlusal outline is almost square, it is slightly wider than long (Tab. 2). The labial cones are drop-shaped and continuous with protoloph and metaloph, respectively. The lingual cones are elongated. Four of the five transverse lophs are long (protoloph, mesoloph, metaloph and posteroloph), only the anteroloph is of medium length. The 1^st^ syncline is narrower than the 2^nd^, 3^rd^ and 4^th^ syncline. The sinus is directed anteriorly, it does not extend to the median line of the tooth. The longitudinal crest is curved, constricted anterior to the mesoloph, and loosely contacts the base of the protocone.

Description of the paratypes and additional material

#### Upper dentition

The labial cones of the upper D4 and M1-M2 are droplet, and continuous with protoloph and metaloph, respectively. The protocone and hypocone (D4, M1, M2) are elongated in antero-labial direction. The anterior arm of the protocone connects with the protoloph and the anteroloph (D4, P4, M1–M3). In D4, P4 and M1 (ZIN 106449) the longitudinal crest is constricted anterior to the mesoloph. The anterior arm of the hypocone connects with the metaloph, and is continuous with the longitudinal crest. The posterior arm of the hypocone is continuous with the posteroloph. All upper teeth have a deep lingual sinus and four labial synclines. In most cases the synclines are closed labially.

**D4** (Fig. 6a) is trapezoidal in occlusal outline, with a longer labial and a shorter lingual part. The tooth-crown is very low. The valleys are wide, the transverse lophs long and low, except for the short anteroloph. A very small 1^st^ syncline is surrounded by the anteroloph, paracone and the protoloph. The mesoloph is long and reaches the labial margin of the D4.

**P4** (Fig. 6b) is rounded in occlusal outline. P4 is as long as wide, it is widest in its anterior part. P4 is higher than D4. The synclines are V-shaped, deep and narrow. The anteroloph is of medium length, however, it is longer than the anteroloph of D4. Its lingual end is fused with the protoloph and with the oblique anterior arm of the protocone. Labially the anteroloph attaches the base of the paracone. The closed 1^st^ syncline is of medium length. The mesoloph of P4 does not reach the labial margin of the tooth. Lingually it attaches the mesocone, labially it contacts the posterior base of the paracone. The hypocone of P4 is reduced. There is no continuous longitudinal crest. Anterior and posterior to the mesocone the sinus is continuous with the 2^nd^ and 3^rd^ syncline, respectively.

**M1** (Fig. 6d) shares the dental pattern with the holotype but differs in two characters: there is no strong connection of protocone and the longitudinal crest, hence, the sinus and 2^nd^ syncline are continuous, and a posterior mesoloph-spur contacts the middle part of the metaloph. The 1^st^, 3^rd^ and 4^th^ synclines are labially closed.

**M2** (Fig. 6e) resembles the morphology of the holotype, however, it differs by smaller size, the shorter mesoloph, the shallow sinus and rather bunodont cones. The 1^st^, 2^nd^ and 4^th^ synclines are closed.

**M3** (Fig. 6f) is smallest. It also has five transverse lophs, four synclines and the lingually closed sinus.

#### Lower dentition

A fragmentary anterior part of the right mandible without teeth (ZIN 106451) is preserved. The occlusal view shows two alveoli of p4 and four alveoli of m1 (Fig. 6m).

**m1/2** (Tab. 2, Fig. 6g, h, i, j, k): The m1 and m2 are similar in characteristics and size and cannot be distinguished with confidence. The lower molars have five transverse lophids, which are continuous with the lingual and labial conids. The lingual conids are higher than the labial ones. Moreover, the metaconid and mesolophid are connected by the short entolophid along of the lingual margin, hence, the 2^nd^ synclinid is closed. The anterolophid is very thin and consists of a labial and a lingual branch (five of seven specimens). It has a lose lingual connection with the base of the metaconid, and labially it contacts the anterior base of the protoconid. The lingual connection of the posterolophid and entoconid is weak but present, hence, the 4^th^ synclinid is also closed. The metalophid has a labial connection with the protoconid. The hypolophid connects with the anterior arm of the hypoconid, and with the short longitudinal crest (in three specimens). The sinusid is deep and directed backward. In two of five m1/2 the longitudinal crest is interrupted, hence, sinusid and 3^rd^ synclinid are fused and extend to the lingual margin of the tooth. The 3^rd^ synclinid is always lingually open, all other synclinids are closed.

**m3** (Fig. 6l): The molar pattern resembles m1/2, but the tooth is smaller, and narrows in posterior direction.

**Root numbers:** Upper teeth (D4, P4 and M1-3) have three roots, one lingual and two labial. The lower p4 has two roots, one anterior and one posterior. The m1–2 have four roots, two anterior and two posterior. The m3 has three roots, two anterior and one posterior.

### Discussion

*Keramidomys* was first described from Europe, later also from China, Mongolia and Siberia, The genus is thought to have originated in East Asia, and from there dispersed to Europe and northwards (Mein 2009; Kimura et al. 2019, 2020).

*K. thaleri*, the oldest and smallest European species is very well represented in numerous faunas of the Neogene mammal Zone MN5 around the Early/Middle Miocene transition. The main diversification and dispersal of European *Keramidomys* species occurred during the Middle and Late Miocene (Fig. 7).

The oldest *Keramidomys* evidence (*K. fahlbuschi*) in Asia is known from the faunas of Aoerban (L) (Early Miocene; Xiejian LMS/A) and Gashunyinadege (Early Miocene; Shanwangian LMS/A) of Nei Mongol in China (Kimura et al. 2020). There, *K. fahlbuschi* co-occurred with other Eomyidae: *Asianeomys*, *Ligerimys, Leptodontomys* and *Pentabuneomys* (Qiu and Li 2016*).* Younger occurrences are known from Loc. 346 and Moergen II (Middle Miocene; Tunggurian LMS/A) and the faunas of Balunhalagen, Huitenghe and Bilutu (Late Miocene; Bahean to Baodean LMS/A (Qiu and Li 2016). Some additional, but not specified occurrences from China and Mongolia are *K.* sp. from the Junggar Basin (China, Early Miocene) and *K.* sp. from Ulan Tolgoi (Valley of Lakes, Mongolia, Early? or Middle Miocene, Mongolian biozone D1/2).

The first finds of *Keramidomys* from the Tagay-1 section in Siberia (Daxner-Höck et al. 2013) were mandibular teeth, combining some dental characteristics of the European *K. mohleri* Engesser, 1972 and the Asian *K. fahlbuschi* Qiu,1996. At that time the diagnostic upper teeth were unknown, hence, the fossils were provisionally described as *K.* aff. *mohleri* vel *K. fahlbuschi*. The according upper D4, P4, M1-M3 were found in the course the excavations in 2014. They show some primitive characteristics (e.g. the anteroloph and 1^st^ syncline of P4 and incomplete lophodonty) reminiscent of *Asianeomys fahlbuschi* Wu et al., 2006 from Aoerban (L) (earliest Miocene; Xiejian LMS/A; Qiu and Li 2016: Fig. 63B). They also share some archaic characters with the oldest occurrences of *K. fahlbuschi*, such as the long, free ending mesoloph, and the characteristic connection between the lingually turned longitudinal crest and the posterior base of protocone of M1. These two characters are predominantly known from Early Miocene (Xiejian and Shanwangian LMS/A), rarely from Middle to Late Miocene occurrences of *K. fahlbuschi* (Qiu and Li 2016: Figs. 65-66). The morphological differences from all European and Chinese species, are sufficient to describe *K. sibiricus* nov. spec. from the Tagay-1 section. The new species from Tagay suggests an early evolutionary stage of *Keramidomys*, and likely an age around the Early/Middle Miocene transition.

## Conclusions

Sediment samples from the middle and upper part of the Tagay-1 section, layers 11, 10, 9, 7, 6, 5 and 3, yielded fossil remains of snails and/or ectothermic vertebrates, and bones and teeth of small mammals. The small mammal record from ~ 2000 kg sediment is poor in terms of specimen numbers, but the diversity of taxa is rather high. Moreover, mammal compositions of the fossil layers (11–5) are in good agreement. They suggest a rather short period of sedimentation. The represented rodent (sub-)families are: Aplodontidae, Mylagaulidae, Sciuridae, Gliridae, Eomyidae, Cricetodontinae and Castoridae. Among them, the glirid Myomiminae indet. and the eomyids *Leptodontomys* cf. *gansus* and *Keramidomys sibiricus* nov. spec. are described in the present paper.

The Gliridae remains are too scarce for reliable genus and species identification. Originating from the Oligocene ancestor group *Gliravus–Peridyromys* manifold Myomiminae genera developed in Europe and Asia Minor during the Early and Middle Miocene. Only a few genera dispersed to eastern and northern Asia.

The Eomyidae from Tagay are two small sized species. The poorly known *L*. cf. *gansus*, which indicates the occurrence of *Leptodontomys* in Siberia, and the well represented *K. sibiricus* nov. spec. The latter differs from all European *Keramidomys* species by some archaic tooth characteristics reminiscent of *Asianeomys*, an Asian Eomyidae genus, that survived from the Oligocene to the Early Miocene. *K. sibiricus* nov. spec. suggests an Asian origin. The assumed age is the Early/Middle Miocene transition.

## Data Availability

All data generated or analysed during this study are included in this published article.

## Abbreviations

NHMW: collection of the Natural History Museum, Vienna (Austria)
ZIN: collection of the Zoological Institute of Russian Academy of Sciences (ZIN), St. Petersburg.
P – M: premolars and molars of the upper dentition
p – m: premolars and molars of the lower dentition
I – i: upper and lower incisors
n: number of specimens
MN: European Neogene Mammal Zone (Steininger 1999)
LMS/A: Chinese Land Mammal Stage/Age (Qiu et al. 2013)
Ma: Million years
Ko: fossils collected by Kossler in the 1990s
